# Supplementary material for: Genetic algorithm with a new round-robin based tournament selection: Statistical properties analysis
Source: PLoS One. 2022 Sep 9;17(9):e0274456. doi: 10.1371/journal.pone.0274456 (PMC9462581; doi:10.1371/journal.pone.0274456)
Supplement: Attachment — Submitted filename: Response of Comments.pdf [file pone.0274456.s002.pdf]

Authors' Response to Reviewers' Comments

Dear Editor-in-Chief,
PLOS ONE

We express our sincere thanks for giving us the opportunity of revising our manuscript entitled "**Genetic algorithm with a new round-robin based tournament selection: statistical properties analysis**" with Manuscript # PONE-D-22-05986. We also appreciate anonymous reviewer(s) and associate editor for providing us their learned input. As per advice, the original manuscript has been revised carefully and the queries are answered to the best of our knowledge. The newly added materials are highlighted by red text.

Below, we provide our responses to the queries posed by the reviewers.

Response to the comments of Academic Editor

Query 1. Please ensure that your manuscript meets PLOS ONE's style requirements, including those for file naming. The PLOS ONE style templates can be found at following links.

Reply. The suggested template has been used in this revised version.

Query 2. Please update your submission to use the PLOS LaTeX template. The template and more information on our requirements for LaTeX submissions can be found at following link.

Reply. The submission has been updated.

Query 3. In your Data Availability statement, you have not specified where the minimal data set underlying the results described in your manuscript can be found. PLOS defines a study's minimal data set as the underlying data used to reach the conclusions drawn in the manuscript and any additional data required to replicate the reported study findings in their entirety.

Reply. The Data Availability statement has been added in updated version and the link is provided as well.

Response to the comments of Reviewer #1

Query 1. Subtopic 4.1 - line 1 - the citation for author "Holland" incomplete (i.e., missing year).

Reply. The missing citation has been cited in this revised manuscript.

Query 2. Subtopic 4.2 - add short-form (SUS) after the title.

Reply. The manuscript has been revised as per suggestions.

Query 3. Subtopic 4.2 - line 1 - the citation for author "Baker" incomplete (i.e., missing year) and revise the whole sentence.

Reply. This missing citation has also been cited and the mentioned sentence has been revised.

Query 4. Please refer to the attachment for correction on minor grammatical errors.

Reply. The corrections have been applied as per suggestions.

Response to the comments of Reviewer #2

Query 1. I suggest more sufficient interpretation to the statistical analysis.

Reply. The manuscript has been revised carefully as per suggestions and more interpretations have been added in Sections 4.4 and 5.3 with computational time is added as well.

Query 2. I suggest sufficient comparison between literature and the manuscript, especially in results.

Reply. In Table 6, computational time is added in this revised version and the proposed operator is compared with all those selection operators which are well known and most used in literature. The TSP is also a well known NP-hard problem and still has its own significant to compare the the new operator of GAs.

Response to the comments of Reviewer #3

Query 1. The contribution is missing. The authors should add a paragraph in the introduction section to clearly describe the contribution of the work.

Reply. The manuscript has been revised and the contribution of the work is added in the second last paragraph in introduction section.

Query 2. The literature review is not sufficient. Most cited references are published before 2016. The latest literature year is the previous work of the author. The authors need to track the latest progress.

Reply. The literature review has been revised as per suggestions in the updated version.

Query 3. Table 6 only analyzes the statistics. But the computational costs are also important. The authors need to compare the minutes of run-time of the proposed method to that of the compared methods.

Reply. Computational costs have been added in this revised version of the manuscript. The Table 6 results are slightly different (because of simulation) from the previous version results.

Finally, we hope our amendments and justifications would achieve your acceptance. Awaiting for your kind reply.

Sincerely yours
Manuscript Authors
